# Tin(IV) Oxide Electron Transport Layer via Industrial-Scale Pulsed Laser Deposition for Planar Perovskite Solar Cells

**DOI:** 10.1021/acsami.3c04387

**Published:** 2023-06-27

**Authors:** Kassio P. S. Zanoni, Daniel Pérez-del-Rey, Chris Dreessen, Nathan Rodkey, Michele Sessolo, Wiria Soltanpoor, Monica Morales-Masis, Henk J. Bolink

**Affiliations:** † Instituto de Ciencia Molecular, Universidad de Valencia, C/Catedrático J. Beltrán 2, 46980 Paterna, Spain; ‡ MESA+ Institute for Nanotechnology, University of Twente, Enschede 7500 AE, The Netherlands

**Keywords:** tin(IV) oxide, electron transport layer, pulsed laser deposition, perovskite solar cell

## Abstract

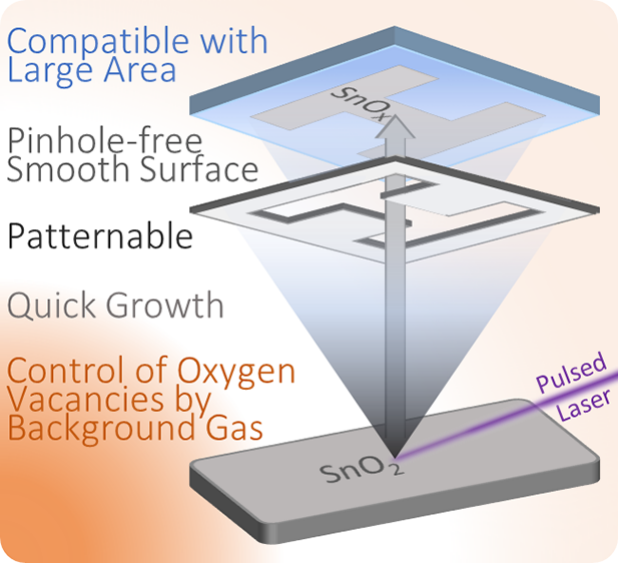

Electron transport layers (ETL) based on tin(IV) oxide (SnO_2_) are recurrently employed in perovskite solar cells (PSCs) by many deposition techniques. Pulsed laser deposition (PLD) offers a few advantages for the fabrication of such layers, such as being compatible with large scale, patternable, and allowing deposition at fast rates. However, a precise understanding of how the deposition parameters can affect the SnO_2_ film, and as a consequence the solar cell performance, is needed. Herein, we use a PLD tool equipped with a droplet trap to minimize the number of excess particles (originated from debris) reaching the substrate, and we show how to control the PLD chamber pressure to obtain surfaces with very low roughness and how the concentration of oxygen in the background gas can affect the number of oxygen vacancies in the film. Using optimized deposition conditions, we obtained solar cells in the n–i–p configuration employing methylammonium lead iodide perovskite as the absorber layer with power conversion efficiencies exceeding 18% and identical performance to devices having the more typical atomic layer deposited SnO_2_ ETL.

## Introduction

Perovskite solar cells (PSCs) have attracted great attention in the past decade as a viable source of efficient and affordable energy, with rapidly increasing power conversion efficiencies (PCEs) since the discovery that they function without acceptor material in 2012.^1^ Such remarkable efficiencies are due in part to the advantageous properties of perovskites: high absorption coefficient, high dielectric constant, high ambipolar charge mobilities, and long charge-carrier diffusion lengths.^2^ The efficient extraction of photogenerated charge carriers to the appropriate electrode relies on the use of proper materials deposited in thin films. This can be achieved using efficient selective charge transport layers in the device configuration.^3,4^

Tin(IV) oxide (SnO_2_) is recurrently used as an electron transport layer (ETL) in perovskite solar cells as its conduction band is well aligned with many perovskite absorbers. Moreover, it demonstrates good charge mobility and can form good contacts with ITO.^4−10^ SnO_2_ also exhibits excellent chemical stability and hence in an ideal device architecture is capable of providing good long-term stability.^11−14^ In n–i–p solar cell architectures, in which the cells are built up in the sequence electron extraction layer (n), perovskite absorber (i), and hole extraction layer (p), SnO_2_ has very low parasitic absorption due to its wide bandgap (3.6–4.1 eV).^10,15^

During the deposition of amorphous SnO_2_, oxygen deficiency can create point defects that lead to shallow subgap states near the conduction band edge capable of trapping free electrons, limiting their mobility and decreasing the conductivity as well as increasing parasitic absorption.^15−19^ Therefore, a fine-tuning of oxygen vacancies (V_Ö_, in accordance with the Kröger–Vink notation^20^) is required for a proper trade-off between optical and electronic properties. Tuning of oxygen vacancies is possible by tuning the background gases (Ar and oxygen) during deposition, leading to a controlled stoichiometry of the SnO_*x*_ films (*x* ≤ 2). As addressed in many recent reviews,^10,14,21,22^ in perovskite solar cells, SnO_*x*_ ETLs have been deposited using many different techniques, from chemical depositions, such as chemical bath, sol–gel, and atomic layer deposition (ALD), to physical depositions, such as magnetron sputtering, thermal evaporation, and pulsed laser deposition (PLD).

In particular, PLD allows to tune the optoelectronic characteristics by controlling the chamber pressure and introducing oxygen or an inert gas in the vacuum chamber.^23−27^ Besides, PLD is a scalable, high throughput manufacturing technology that can produce conformal compact films on flat and textured surfaces and allow for precise control of thickness. Moreover, recently we demonstrated that PLD is a method that allows for soft deposition of metal oxide film without damaging underlaying organic or perovskite based semiconductors.^26,27^ Despite its encouraging perspectives, there is only one example in the literature of PLD being employed to deposit SnO_2_ ETLs, in which Chen et al. demonstrated that the amorphous nature of the films deposited by PLD is suitable for flexible photovoltaics.^28^ In that work, an average PCE of 16.3% was obtained, yet the devices suffered from fairly high series and low shunt resistances due to the low conductivity and slightly rough surface of the SnO_2_ films, respectively, which limited the fill factor (FF) to 70% on average.

Herein, we report on the effects of the chamber pressure and the oxygen partial pressure on the optoelectronic characteristics of amorphous SnO_*x*_ films deposited by using an industrial PLD tool and its use as an ETL to achieve high-efficiency perovskite solar cells. Operating at room temperature, we optimized the deposition parameters to mitigate subgap states and hence improve the conductivity of the SnO_*x*_ layer. The PLD tool is equipped with a droplet trap that reduces the number of undesired particles on the deposited film. As a result, we obtained smooth surfaces with nanometric roughness, which is crucial to improve the rectification of the current density–voltage (*J*–*V*) curve in evaporated planar PSCs. Using this optimized PLD-SnO_*x*_ ETL, we achieve planar MAPbI_3_ solar cells in the n–i–p configuration (in which MAPbI_3_ is methylammonium lead iodide perovskite) with power conversion efficiencies exceeding 18%.

## Results and Discussion

### Deposition Conditions

The SnO_*x*_ deposition conditions were optimized by using a constant laser fluence of 1.5–1.6 J cm^–2^, with a laser frequency set at 25 Hz. The substrates were kept at room temperature during the whole PLD process. Chamber pressures (*P*_chamber_) on the order of 10^–3^ mbar were employed, which are known to ensure flat, virtually pinhole-free films in comparison to depositions at increased pressures with gradually increasing porosity and granular structures.^27^ The oxygen partial pressures during deposition were controlled by a constant injection of an oxygen/argon gas mixture with the desired concentrations. More specifically, we tested four different conditions: three samples at a *P*_chamber_ of 5.0 × 10^–3^ mbar with increasing contents of O_2_, i.e., 40% (60% Ar), 70% (30% Ar), and 100%, and a fourth sample with a higher *P*_chamber_ of 8.0 × 10^–3^ mbar and 100% O_2_. For all of the optoelectronic characterizations and device fabrications, the deposited SnO_*x*_ films had a final thickness of 20 nm. Under these deposition conditions, the growth rate of the films was 120 nm/h on average. This is much faster than a typical ALD process for the SnO_*x*_ deposition which is in the range 5.0–10.0 nm/h in a process that requires heating of the chamber to 90 °C and of the gas inlet manifolds to 150 °C. The PLD SnO_*x*_ layers were annealed at 150 °C for 30 min in a N_2_ glovebox directly after the PLD deposition as this typically increases the film conductivity.^15^ To verify if the annealing has an impact on the solar cell performance, we compared two cells, one with an annealed SnO_*x*_ and one with a SnO_*x*_ film that was not annealed, and the former showed a better performance as shown in Figure S1 of the Supporting Information.

### Characterization of SnO_*x*_ Thin Films

**Figure 1:**
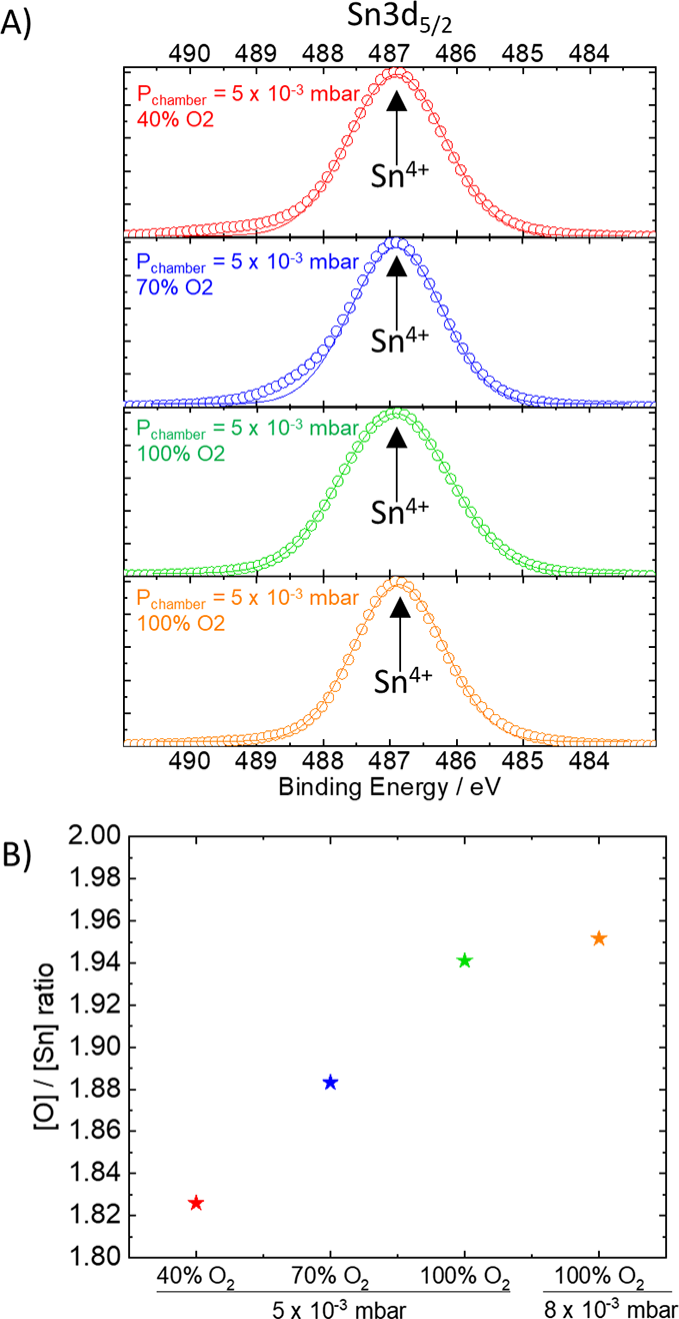
(A) XPS Sn 3d_5/2_ peaks (in circles) with deconvoluted components (solid lines) and (B) atomic compositions of annealed SnO_*x*_ layers (20 nm) deposited under different PLD chamber pressures and oxygen concentrations.

As depicted in Figure 1A, X-ray photoelectron spectroscopy (XPS) confirmed the 4+ oxidation state of the Sn ions in our samples, with the typical Sn^4+^ 3d_5/2_ peak centered around 486.8 eV (the full spectrum and fittings are shown in Figure S2). Note that we did not observe the peak of Sn^2+^ or metallic Sn^0^ usually found around 486 and 485 eV, respectively,^29,30^ suggesting no degradation during the PLD deposition. XPS also revealed lower amounts of oxygen in the atomic composition of the developed SnO_*x*_ films as the oxygen partial pressure during the PLD process decreased (Figure 1B). Therefore, the empirical formulas of the SnO_*x*_ films deposited under 100% (at both employed *P*_chamber_ pressures), 70%, and 40% O_2_ concentrations are estimated as SnO_1.95_, SnO_1.88_, and SnO_1.83_, even though the composition of the target employed is a stoichiometric SnO_2_. This difference in O_2_ content in the deposited films is due to the exchange of O_2_ from the target with the background gas as previously demonstrated by Morales-Paliza et al.^23^ Therefore, a lower O_2_ partial pressure during deposition leads to a lower retention of oxygen into the bulk of the film, ultimately generating increasing amounts of oxygen vacancies (V_Ö_).

Similar to other reported room temperature PLD processes,^27,28^ the pristine PLD-SnO_*x*_ films were amorphous, with no peaks to be found in the X-ray diffraction (XRD) pattern (Figure S3). Moreover, even though annealing can be expected to enhance crystallinity of amorphous films, the XRD of the annealed SnO_*x*_ films also revealed an amorphous nature, as similarly observed by Rucavado et al. for Zn-doped SnO_2_ annealed at 500 °C.^15^

**Figure 2:**
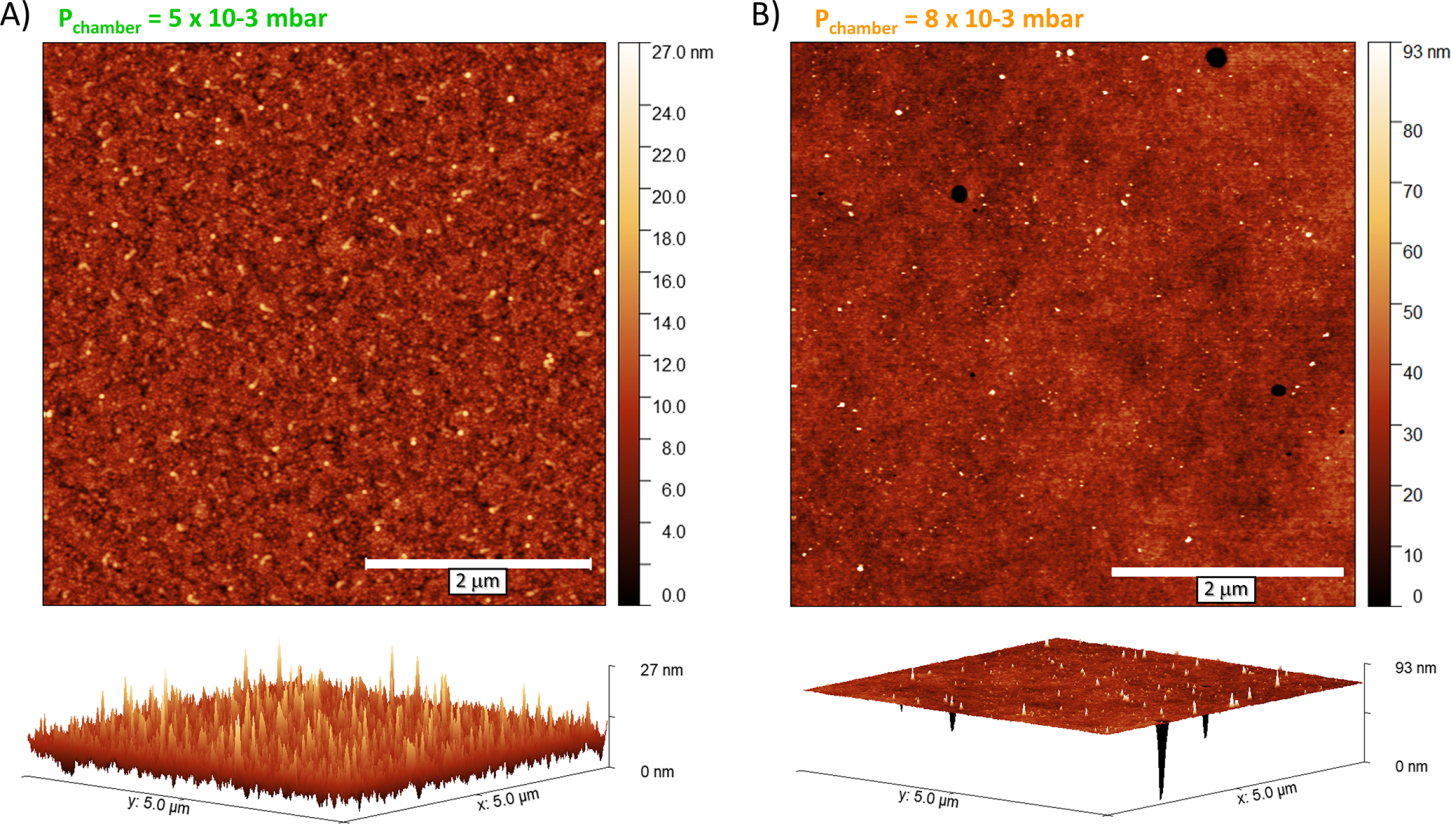
AFM surface profiles of annealed SnO_*x*_ films deposited by PLD on glass with a *P*_chamber_ of 5.0 × 10^–3^ mbar (A) or 8.0 × 10^–3^ mbar (B).

Atomic force microscopy (AFM) of the 20 nm SnO_*x*_ films on glass (Figure 2) revealed very flat surfaces for both films, yet the one deposited at *P*_chamber_ at 5.0 × 10^–3^ mbar shows an RMS of around 2.2 nm, homogeneous throughout the whole 5 × 5 μm^2^ area, while the one deposited at 8.0 × 10^–3^ mbar shows RMS variations from 1.2 nm in the larger area to 7.1 nm in a few smaller regions, with structures that resemble pinholes. Moreover, the presence of occasional undesired particles or debris was not detected in any of the samples as our PLD tool is equipped with a droplet trap to prevent them.

**Figure 3:**
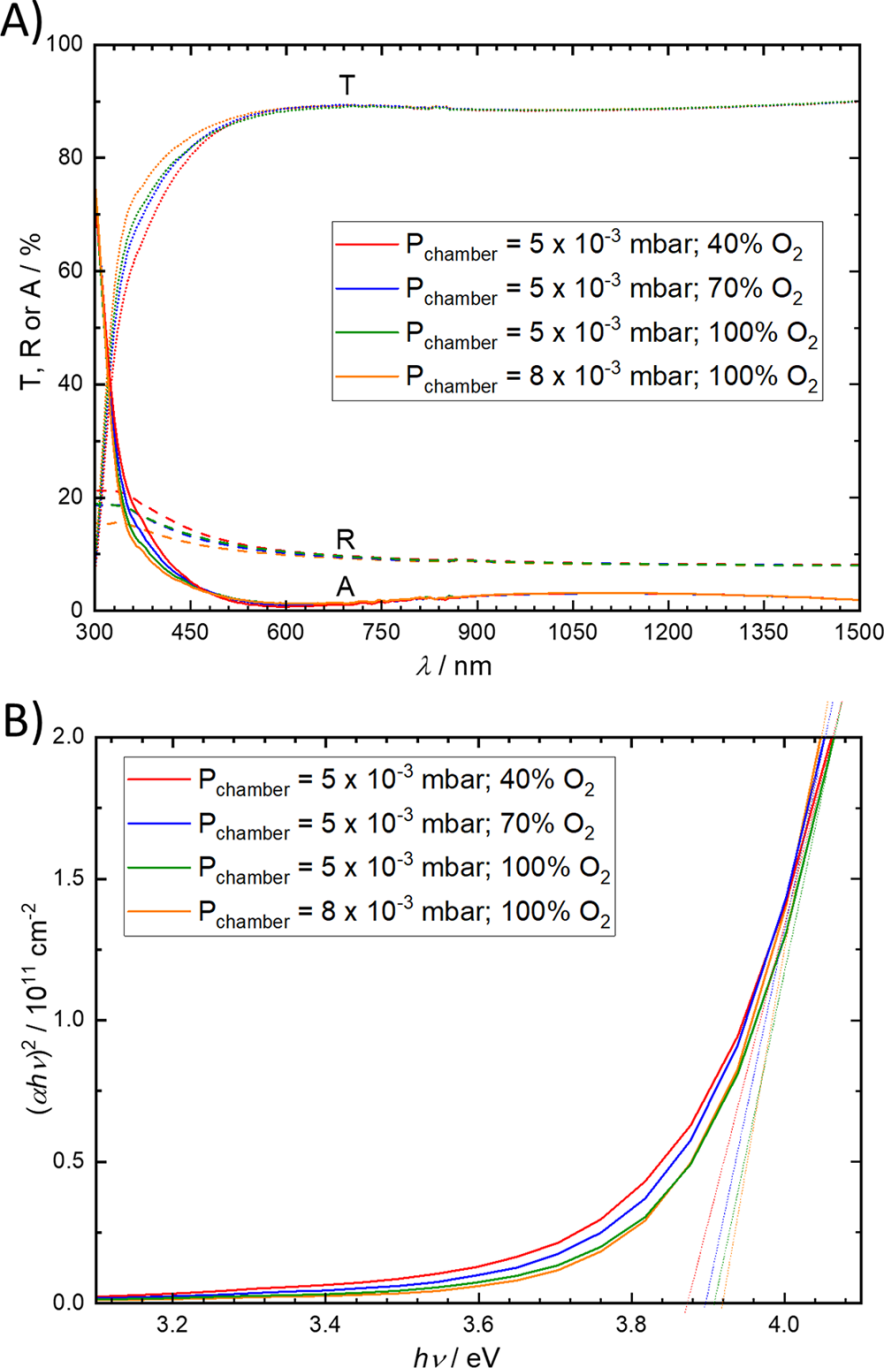
(A) Transmittance (*T*, ···), reflectance (*R*, ---), and absorptance (*A*, −) spectra. (B) Tauc plot of annealed SnO_*x*_ layers (20 nm) deposited under different PLD chamber pressures and oxygen concentrations.

The transmittance (*T*), reflectance (*R*), and absorptance (*A* = 1– *T* – *R*) spectra of the 20 nm SnO_*x*_ films on glass are depicted in Figure 3. All samples have a high *T* in the whole visible and near-infrared spectrum (*T* ≈ 90%), regardless of *P*_chamber_ or the O_2_ concentration, with losses coming mainly from a weak *R* of ∼10% in the whole spectral range. Because SnO_*x*_ is a wide bandgap semiconductor, it absorbs light strongly in the ultraviolet region, at wavelengths lower than 320 nm. Based on the Tauc plots, the optical bandgap energies were estimated to be around 3.90 ± 0.01 eV. Additional weak absorptions are observed between 320 and 400 nm, and these absorptions increase proportionally with the amount of V_Ö_ in the films. We therefore ascribe these to the absorption arising from subgap states, as similarly observed elsewhere.^15,31^

Despite the occasional subgap states from V_Ö_, Kelvin probe measurements indicate a similar work function of ∼4.7 eV for all of the investigated samples, regardless of chamber pressure and oxygen partial pressure (Figure S4). This value is in accordance with other reported one for SnO_2_ films.^32,33^ Their similar work functions should lead to similar band alignments and Ohmic contacts when these PLD SnO_2_ films are applied in perovskite solar cells; therefore, any changes in device performance should be accounted for by other factors, for example, the presence of V_Ö_ subgap states.

**Figure 4:**
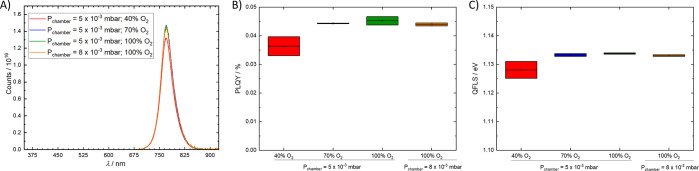
(A) Photoluminescence spectra, (B) PLQY, and (C) QFLS of MAPbI_3_ perovskite on glass/ITO/SnO_*x*_ stacks having SnO_*x*_ layers (20 nm) deposited under different PLD chamber pressures and oxygen concentrations.

To evaluate if the V_Ö_ subgap states of the deposited SnO_*x*_ films can introduce more nonradiative recombination to the perovskite absorber, stacks consisting of glass/ITO/SnO_*x*_/MAPbI_3_ were deposited, and the steady-state photoluminescence (PL) was determined. As is typical of MAPbI_3_, all samples exhibited a narrow emission band around 770 nm (Figure 4A). As summarized in Figure 4B, the PL quantum yields (PLQY) of the perovskite films with the SnO_*x*_ films deposited at high O_2_ concentrations (i.e., with low V_Ö_) are similar among each other (PLQY ≈ 0.045%); however, the perovskite emission got slightly quenched by the SnO_*x*_ deposited at the lowest O_2_ concentration (i.e., with the highest V_Ö_), reducing the PLQY to 0.037%. This is ascribed to an increased nonradiative recombination through extracted electrons via the V_Ö_ subgap states, which in turn decrease the density of free photogenerated electrons (or quasi-Fermi level splitting, QFLS = *V*_OC,rad_ + *kT* ln(PLQY), Figure 4C) in the perovskite.^34^

We have tried to measure the sheet resistance of our SnO_2_ thin films either via measurement methods such as van der Pauw or four-probe measurements, but their sheet resistances were beyond the limit of detection of our equipment (>10^6^ Ω/□). It is known that the resistance of undoped oxide semiconductors is very high; for example, Chen et al. have published values as high as 10^8^ Ω/□ for SnO_2_ thin films similarly deposited by PLD at room temperature,^28^ in accordance with our own observations. What is typically done instead to obtain information about the conductive properties is to measure the series resistances of devices employing these semiconductor materials, which will be presented in the next section.

### SnO_*x*_ ETL in Perovskite Solar Cells

**Figure 5:**
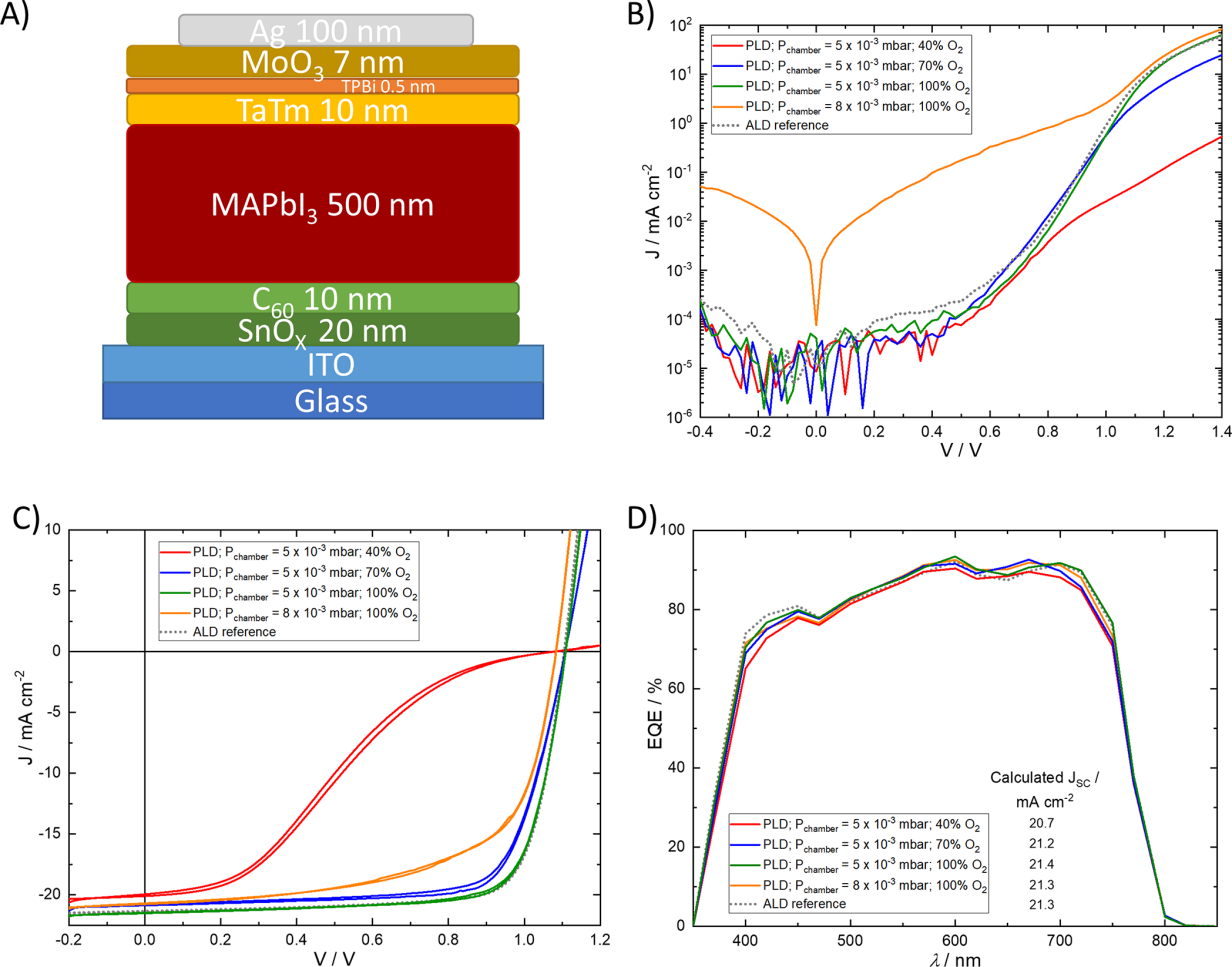
(A) Schematic device architecture, (B) dark *J*–*V* curves, (C) external quantum efficiency (EQE) spectra, and (D) illuminated *J*–*V* curve (measured under AM 1.5 G irradiation at 100 mW cm^–2^ at room temperature; forward and reverse scans are presented) of ITO/SnO_*x*_/C_60_/MAPbI_3_/TaTm/TPBi/MoO_3_/Au devices with SnO_*x*_ ETL deposited under different PLD chamber pressures and oxygen concentrations. The represented curves are the average curves of at least 16 samples.

The developed SnO_*x*_ films were tested as the ETL in solar cells in the n–i–p configuration, as represented in Figure 5A. This consisted of a glass substrate followed by these layers: ITO (130 nm)/SnO_*x*_ (20 nm)/C_60_ (10 nm)/MAPbI_3_ (500 nm)/TaTm (10 nm)/TPBi (0.5 nm)/MoO_3_ (7 nm)/Au (100 nm) (where C_60_ is fullerene, TaTm is N4,N4,N4″,N4″-tetra([1,1′-biphenyl]-4-yl)-[1,1′:4′,1″-terphenyl]-4,4″-diamine, TPBi is 2,2′,2″-(1,3,5-benzinetriyl)-tris(1-phenyl-1*H*-benzimidazole), and MoO_3_ is molybdenum(VI) oxide). For comparison, reference devices were also fabricated where SnO_*x*_ was deposited using ALD instead of PLD (see the Experimental Section for deposition details). The employed architecture relied on C_60_ and TaTm as intrinsic organic materials for charge selection and SnO_*x*_ and MoO_3_ as the charge transport layers for efficient extraction of the photogenerated charge carriers to the external circuit.^35^ Without the thin C_60_ layer in between the SnO_*x*_ and the evaporated perovskite film, we obtained poor fill factors (see the *J–V* curve of a device, e.g., without C_60_ in Figure S5). This is in line with our previous observation on solar cells employing solution processed TiO_2_ charge extraction layers. In that work, very poor performances were obtained when the evaporated perovskite film was deposited directly on the TiO_2_ layer. This led to poor fill factors and low power conversion efficiency. The insertion of a thin C_60_ layer alleviated this and led to solar cells reaching PCE’s of 20%.^36^ Moreover, TPBi was employed to protect TaTm from the diffusion of MoO_3_, which enables the use of this oxide in n–i–p configuration without compromising the performance or reproducibility of the device.^35^

The dark *J*–*V* curves for devices containing the developed SnO_*x*_ ETL are shown in Figure 5B. We note that due to the minimum current range limit of our current meter, the region with current densities lower than 10^–4^ mA cm^–2^ (lying around −0.4 to 0.6 V) is poorly resolved. The current density at this voltage range, usually referred as leakage current,^37^ was very similar (despite the lack of resolution) for samples deposited at 5 × 10^–3^ mbar at all the used oxygen contents; however, it was slightly higher for the device with PLD SnO_*x*_ deposited at 8 × 10^–3^ mbar. This implies that the pinholes observed in Figure 3 for this sample act as a direct shunt between ITO and C_60_/perovskite through which the current can leak, leading to parasitic losses in performance when the device is illuminated.^37^ On the other hand, the dark current density around 1.1 V, dictated by the total series resistance (*R*_s_) in the solar cell,^38^ was very similar for the devices having PLD SnO_*x*_ deposited under O_2_ saturation (100%, either at 5 × 10^–3^ or at 8 × 10^–3^ mbar). As such, the calculated *R*_s_ values (Table 1) for these two devices are identical, i.e., 5.8 Ω cm^–2^. However, as the O_2_ content during the PLD deposition decreased first to 70% and then to 40%, the current density at this voltage also decreased because of an increase in *R*_s_ to 11 and 60 Ω cm^–2^, respectively. This indicates that the V_Ö_ subgap states observed for the PLD SnO_*x*_ deposited at low O_2_ concentrations can decrease the conductivity by trapping the electrons passing through. Finally, the global trend of the dark current density (and calculated *R*_s_) for the devices having PLD SnO_*x*_ deposited at 5 × 10^–3^ mbar (100% O_2_) is very similar to that of the reference device that has SnO_*x*_ deposited via ALD.

**Table 1:** Photovoltaic Performance of ITO/SnO_*x*_/C_60_/MAPbI_3_/TaTm/TPBi/MoO_3_/Au Devices with the SnO_2_ ETL Deposited under Different PLD Chamber Pressures and Oxygen Concentrations^a^

SnO_*x*_	*J*_SC_ (mA cm^–2^)	*V*_OC_ (V)	FF (%)	PCE (%)	*R*_s_ (Ω cm^–2^)^b^
PLD; *P*_chamber_ = 5 × 10^–3^ mbar; 40% O_2_	20.2 ± 0.7	1.09 ± 0.01	28 ± 4	5.1 ± 0.9	60 ± 8
PLD; *P*_chamber_ = 5 × 10^–3^ mbar; 70% O_2_	21.0 ± 0.7	1.11 ± 0.01	70 ± 4	16.4 ± 0.8	11 ± 1
PLD; *P*_chamber_ = 5 × 10^–3^ mbar; 100% O_2_	21.4 ± 0.3	1.11 ± 0.01	77 ± 1	18.1 ± 0.6	5.8 ± 0.7
PLD; *P*_chamber_ = 8 × 10^–3^ mbar; 100% O_2_	21.1 ± 0.6	1.09 ± 0.01	65 ± 6	14.9 ± 1.6	5.8 ± 0.7
ALD	21.5 ± 0.3	1.11 ± 0.01	77 ± 1	18.2 ± 0.8	4.0 ± 0.8

a Notation: average ± standard deviation. Number of samples: at least 16 samples for each SnO_*x*_.

b Calculated from the voltage-independent region of the difference of the light and dark *J–V* curves using the method described by Grabowski et al.^39^

The current density versus voltage (*J*–*V* curve) under 1 sun illumination for the investigated devices is depicted in Figure 5C (forward and reverse scans are shown), and the photovoltaic parameters are summarized in Table 1. For devices fabricated with the SnO_*x*_ layer deposited at a chamber pressure of 5 × 10^–3^ mbar under different O_2_ concentrations, the observed changes in *R*_s_ are likely due to the V_Ö_ defect states affecting mostly their fill factor (FF), increasing from 28% to 77% as the O_2_ concentration during SnO_*x*_ deposition increased from 40% to 100%, respectively. Another clear effect is on the hysteresis of the curves; typically, fully evaporated flat solar cells have negligible hysteresis, as similarly observed for the sample with SnO_*x*_ deposited at 100% O_2_ saturation; however, some hysteresis was observed as the O_2_ concentration decreased. The open circuit voltage (*V*_OC_) reached 1.11 V for devices having SnO_*x*_ deposited under saturated O_2_ but decreased slightly to 1.09 V for an O_2_ concentration of 40%. The trend in *V*_OC_ is in line with the one observed for the QFLS.^40^ In a similar fashion, the *J*_SC_ was higher for the solar cell employing the SnO_*x*_ film deposited at 100% O_2_ atmosphere, with 21.4 mA cm^–2^, and decreased gradually for decreasing amounts of O_2_ during the SnO_*x*_ deposition, reaching the lowest value at 20.2 mA cm^–2^ for 40% O_2_. As expected, their external quantum efficiency (EQE, Figure 5D) magnitude follows this trend in *J*_SC_. The integrated current density from the EQE curves leads to similar values as those for the measured *J*_SC_. Even though their EQE profiles were very similar, the device with the SnO_*x*_ ETL deposited at 40% O_2_ showed overall a lower EQE value. This is attributed primarily to a reduced charge extraction following the increased *R*_s_ due to more oxygen vacancies. Optical effects also played a role at wavelengths lower than 400 nm, in which the increased V_Ö_ subgap states led to additional parasitic absorption (see transmittance spectra in Figure 3).

The PSCs employing the PLD-SnO_*x*_ deposited under 8 × 10^–3^ mbar (100% O_2_) showed *J*–*V* curves that were virtually free of hysteresis, and the devices showed similar *J*_SC_ and EQE to the one fabricated at 5 × 10^–3^ mbar (100% O_2_). However, their *V*_OC_ and FF are slightly lower as a possible consequence of the aforementioned shunts, through which the leakage current can flow in parallel to the photocurrent.^37^

The best performance of the PSC was achieved with a SnO_*x*_ layer deposited at 5 × 10^–3^ mbar and 100% O_2_, leading to a PCE of 18.2%. This performance is on par with that of the reference devices fabricated with ALD SnO_*x*_, which is well-known for leading to flat pinhole-free SnO_*x*_ layers and reproducible performances,^10,11,41,42^ proving the quality of our PLD films. The PLD process is much faster than ALD and can be done on larger areas and industrial scales, but perhaps the main advantage over ALD is that PLD allows the use of shadow masks for patterning, which can make the widespread use of SnO_*x*_ ETLs even more appealing.

## Conclusions

In conclusion, we demonstrated that the PLD deposition of SnO_*x*_ electron transport layers should be performed under O_2_-saturated conditions in order to reduce the formation of oxygen vacancies. This leads to lower parasitic absorption and low resistivity. Crucial to avoid shunts or even shortened devices, flat pinhole-free surfaces with nanometric roughness and no debris can be obtained with deposition pressures of 5 × 10^–3^ mbar. Using the optimum SnO_*x*_ film, planar n–i–p perovskite solar cells can reach power conversion efficiencies exceeding 18%, with similar PCE to a reference device employing ALD deposited SnO_*x*_. Because of the superior deposition speed, low material consumption, the possibility to use shadow mask, and proven scalability, the PLD process holds more promise for industrialization.

## Experimental Section

TaTm was provided by TCI. C_60_ was purchased from Sigma-Aldrich. PbI_2_ was purchased from Tokyo Chemical Industry CO. MoO_3_, TPBi, and methylammonium iodide (MAI) were purchased from Lumtec.

Prepatterned ITO-coated glass substrates were purchased from Naranjo Substrates. They were cleaned by subsequent immersions in soap, water, deionized water, and isopropanol in a sonication bath for 5 min each, followed by insertion in an ozone chamber with UV irradiation for 20 min.

### Industrial Scale PLD System

The SnO_*x*_ films were deposited at room temperature using a Solmates large area PLD 200 mm system. This PLD tool was coupled to a N_2_ glovebox to minimize any detrimental effects from the presence of O_2_ and moisture on the performance of the finally produced devices. A Lightmachinery’s IPEX-700 KrF excimer laser (λ = 248 nm) was employed, setting the repetition rate at 25 Hz and a fluence of 1.5–1.6 J cm^–2^. The source material for SnO_2_ deposition was an SnO_2_ ceramic target (99.9%), acquired from Pi-kem. The substrates were allocated 90 mm on top of the target. The system was equipped with a droplet trap to reduce the number of undesired particles on the deposited film, which allowed for a homogeneous deposition on large areas >615 cm^2^, with less than 1.5% variation in TCO thickness and sheet resistance. The droplet trap consisted of a large metal disk, with only four round openings at 90° from each other. The droplet trap was located between the target and the substrates, rotating at a speed of 3000 rpm. The laser was synchronized with the rotation of the disk, so that the laser can hit the target when the opening was directly above the target; in that way, the generated plasma plume can pass through the opening and reach the substrate. Any particle or drop generated slightly later will be blocked by the metal disk as the opening has moved away.

### Device Fabrication

The solar cell layers were prepared by vacuum sublimation of the entire solar cell stack on glass/ITO/PLD-SnO_*x*_ substrates. The substrates were 3 × 3 cm^2^ and contained 16 rectangular working pixels, each pixel with an approximate area of 0.082 cm^2^, with a similar layout to the one published recently;^27^ all the samples were irradiated from the glass side using an illumination mask positioned at the center of the pixels with an opening of precise 0.050 cm^2^. Thermal vacuum deposition was performed in vacuum chambers evacuated to a pressure of 10^–6^ mbar, which were integrated into a nitrogen-filled glovebox (H_2_O and O_2_ < 0.1 ppm). In general, the vacuum chambers were equipped with temperature-controlled evaporation sources (Creaphys) fitted with ceramic crucibles. The sources were directed upward with an angle of approximately 90° with respect to the base of the evaporator. The distance between the substrate holder and the evaporation source was approximately 30 cm. Individual quartz crystal microbalance (QCM) sensors monitored the deposition rate of each evaporation source, and another one close to the substrate holder monitored the total deposition rate. For the perovskite deposition, MAI and PbI_2_ were coevaporated at the same time by measuring the deposition rate of each material in two QCM sensors and obtaining the total perovskite thickness in a third one located closer to the substrates, leading to a 500 nm thick perovskite. TaTm, C_60_, and TPBi were sublimed in the same vacuum chamber with temperatures around 300, 400, and 200 °C, respectively, to a precise desired thickness. MoO_3_ and Ag were evaporated in another vacuum chamber using tungsten boats as sources by applying currents ranging from 2.0 to 4.5 A. For the reference devices, a 20 nm layer of SnO_*x*_ was deposited by ALD following a procedure recently published by us^43^ using an Arradiance’s GEMStar XT Thermal ALD system integrated into a nitrogen-filled glovebox. In summary, the ALD chamber was heated to 90 °C, the bottle containing the Sn precursor (tetrakis(dimethylamino)tin, TDAT) was heated to 60 °C, and the bottle of the oxidizer (water) was not heated; the precursor and oxidizer manifolds were heated to 115 and 140 °C, respectively. Prior to deposition, the tubes and valves in the manifolds were degassed three times by performing a series of 30 pulses with the bottles manually closed. The ALD cycle consisted of consecutive purges of TDAT for 550 ms and water vapor for 200 ms, each followed by N_2_ purges of 30 and 105 s, respectively, to ensure the complete removal of the precursors from the ALD chamber. All the devices showed here, with either PLD or ALD SnO_2_, were encapsulated using ALD of Al_2_O_3_ at 40 °C, using a protocol recently published by us.^44^

### General Characterization

T, R, and A spectra between 300 and 1500 nm were collected using a PerkinElmer Lambda 950 UV–vis–NIR spectrophotometer, coupled with an integrating sphere; by assuming a direct band gap transition and a thickness of 20 nm, the optical band gap of these materials was then estimated using Tauc plot analysis. XPS spectra were recorded using a Thermo Scientific K-Alpha with a monochromatic Al Kα X-ray source (1486.6 eV); data were analyzed and deconvoluted with Avantage software, and the binding energies were adjusted to the standard C 1s peak at 284.6 eV. Thicknesses were measured with an Ambios XP1 mechanical profilometer. X-ray diffraction was measured with a Panalytical Empyrean diffractometer equipped with a Cu Kα anode operated at 45 kV and 30 mA and a Pixel 1D detector in scanning line mode; single scans were acquired in the 2θ = 5°–50° range in Bragg–Brentano geometry in air. AFM of sample surfaces was obtained using a Bruker ICON Dimension microscope in tapping mode. Work function measurements were obtained by using a KP Technology Air Photoemission and Kelvin Probe system.

The external quantum efficiency (EQE) was estimated using the cell response at different wavelengths (measured with a white light halogen lamp in combination with band-pass filters). A possible solar spectrum mismatch was checked with a calibrated silicon reference cell (MiniSun simulator by ECN, from Netherlands) by means of the correction methodology published by NREL.^45^ The *J*–*V* curves for the solar cells were recorded using a Keithley 2612A SourceMeter in −0.2 and 1.2 V voltage range with 0.01 V steps and integrating the signal for 20 ms after a 10 ms delay, corresponding to a speed of about 0.3 V s^–1^. The devices were illuminated under a Wavelabs Sinus 70 AAA LED solar simulator. The light intensity was calibrated before every measurement using a calibrated Si reference diode equipped with an infrared cutoff filter (KG-3, Schott). For all devices, we compared the calculated *J*_SC_ from the EQE with that obtained from the *J*–*V* analysis of the cells illuminated with the AAA Led solar simulator. During experiments, the encapsulated devices were exposed to air, and temperature was stabilized at 298 K using a cooling system controlled by a Peltier element.

## Supporting Information Available

Additional *J*–*V* curves, XPS spectra, and XRD pattern (PDF)
