# Supplementary material for: Tin(IV) Oxide Electron Transport Layer via Industrial-Scale Pulsed Laser Deposition for Planar Perovskite Solar Cells
Source: ACS Appl Mater Interfaces. 2023 Jun 27;15(27):32621–8. doi: 10.1021/acsami.3c04387 (PMC10347112; doi:10.1021/acsami.3c04387)
Supplement: Supplementary file 1 — am3c04387_si_001.pdf [file am3c04387_si_001.pdf]

Supporting Information

Tin(IV) Oxide Electron Transport Layer via Industrial-Scale Pulsed Laser Deposition for Planar Perovskite Solar Cells

Kassio P.S. Zanoni[1,*], Daniel Pérez-del-Rey[1], Chris Dreessen[1], Nathan Rodkey[1], Michele Sessolo[1], Wiria Soltanpoor[2], Monica Morales-Masis[2], Henk J. Bolink[1,*]

[*] Corresponding authors: kassio.zanoni@uv.es; henk.bolink@uv.es
[1] Instituto de Ciencia Molecular, Universidad de Valencia, C/Catedrático J. Beltrán 2, 46980 Paterna, Spain
[2] MESA+ Institute for Nanotechnology, University of Twente, Enschede 7500 AE, The Netherlands

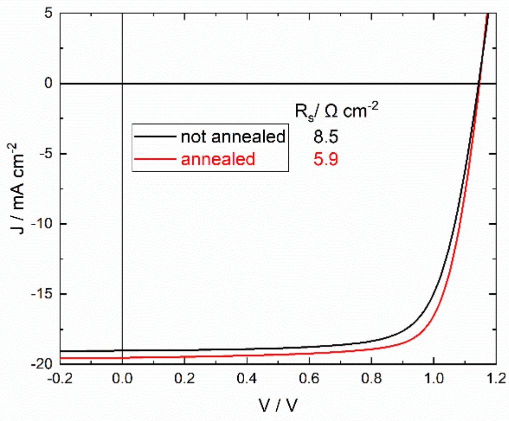

Figure S1 – Illuminated J–V curve (measured under AM 1.5 G irradiation at 100 mW cm^{-2} at room temperature) of ITO/SnO_X/C_{60}/$MAPbI_3$/TaTm/TPBi/MoO_3/Au devices with SnO_2 ETL deposited by PLD at $P_{chamber}$ = 5 x 10^{-3} mbar and 100% O_2, annealed or not annealed at 150 ºC for 30 min inside a N_2 glovebox. The series resistances shown in the figure were calculated from the voltage independent region of the difference of the light and dark JV curves using the method described by Grabowski et al (ref 39 in the manuscript).

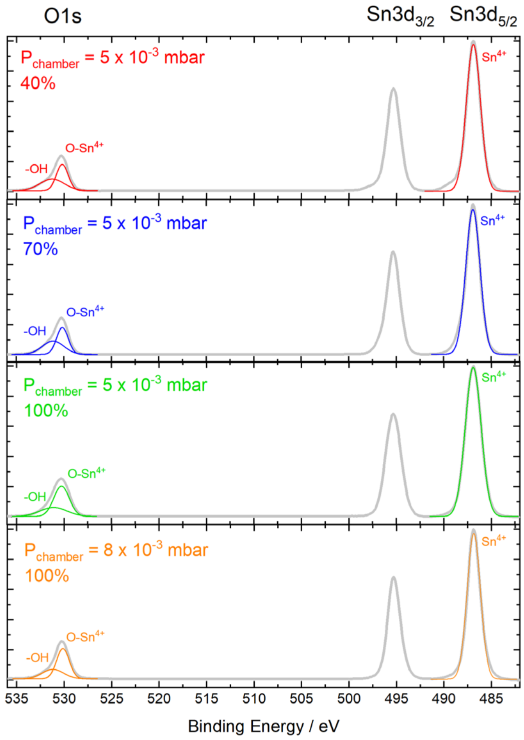

Figure S2 – XPS spectra (grey) with deconvoluted components (colored) of annealed SnO_X layers (20 nm) deposited under different PLD chamber pressures and oxygen concentrations.

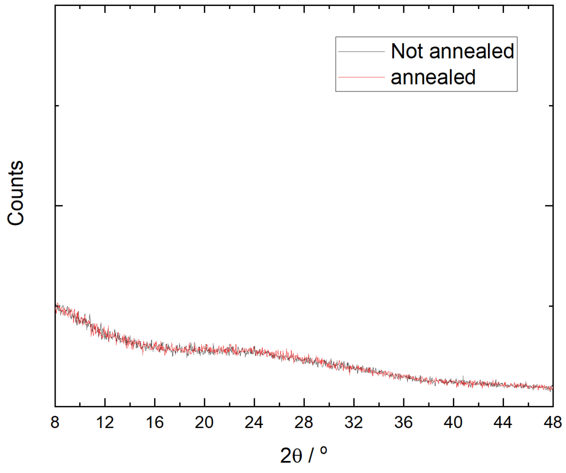

Figure S3 – Amorphous nature of the SnO_2 films deposited by PLD at $P_{chamber}$ = 5 x 10^{-3} mbar and 100% O_2, annealed or not annealed at 150 ºC for 30 min inside a N_2 glovebox, with no peaks to be found on their XRD pattern.

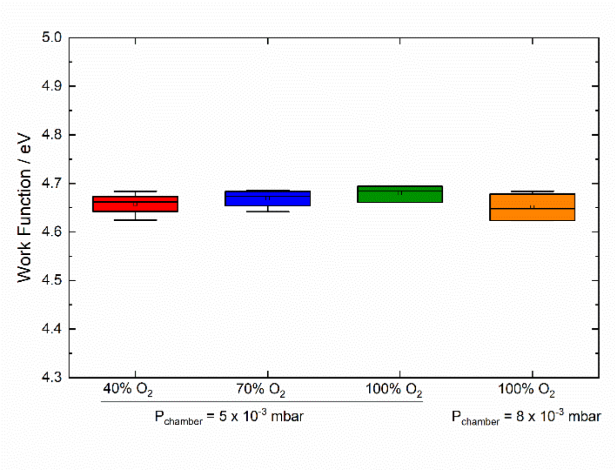

Figure S4 – Work functions of the surface of annealed SnO_X layers (20 nm) deposited under different PLD chamber pressures and oxygen concentrations.

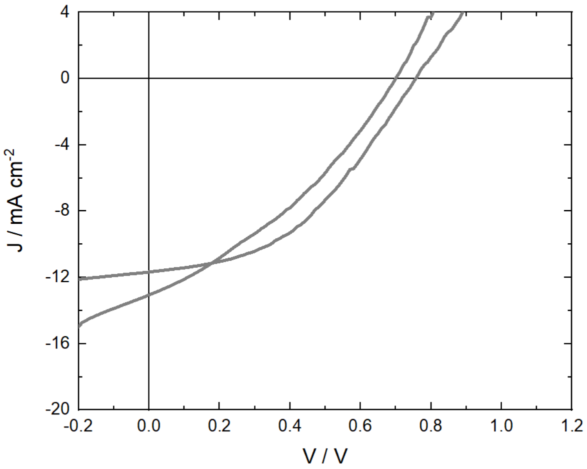

Figure S5 – Average JV curve of ITO/SnO_X/$MAPbI_3$/TaTm/TPBi/MoO_3/Au devices, without C_{60} intrinsic layer between SnO_X and $MAPbI_3$, depicting the device's poor performance.

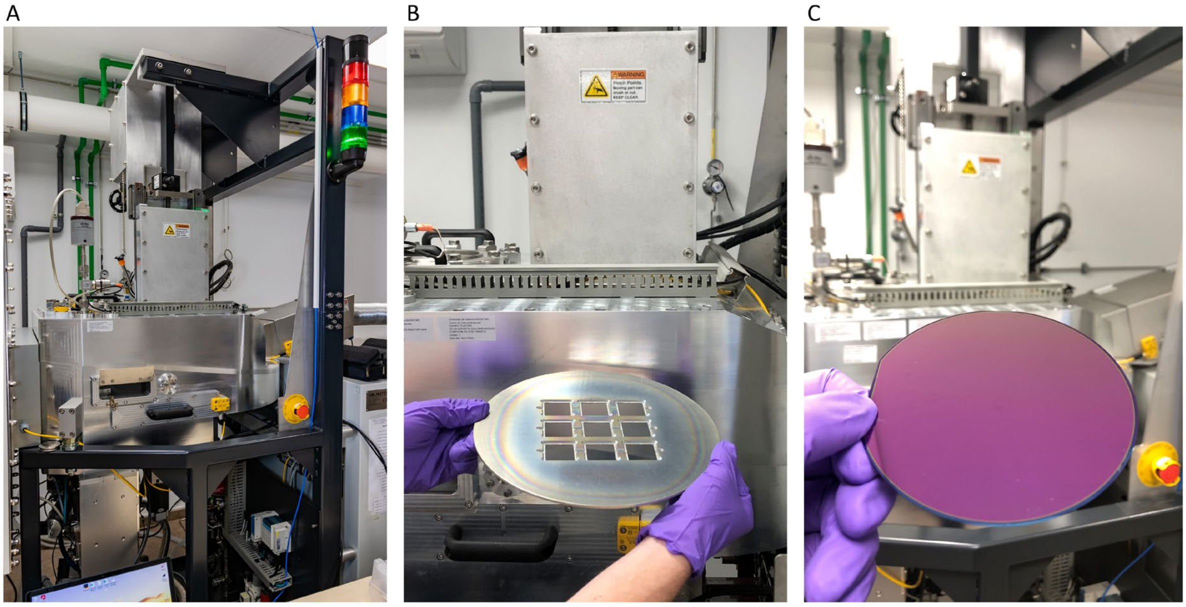

Figure S6 – PLD tool. Panel A, side view of the PLD deposition chamber. Panel B. Substrate holder used for the ITO deposition on the solar cells, the holder consists of a round carrier wafer, with a deposition radius of 125 mm, i.e. an equivalent area of ~490 cm2, that can support up to nine substrates of 3x3 cm2. Panel C. Si-wafer of 80 cm^2 with uniformly deposited ITO film showing less than 1.5% of variation in thickness, roughness and sheet resistance over the whole area.
